# Methods for an International Randomized Clinical Trial to Investigate the Effect of Gsk249320 on Motor Cortex Neurophysiology using Transcranial Magnetic Stimulation in Survivors of Stroke

**DOI:** 10.4172/2167-0870.1000199

**Published:** 2014

**Authors:** Matt P. Malcolm, Lori Enney, Steven C Cramer

**Affiliations:** 1 Integrative Rehabilitation Laboratory, Department of Occupational Therapy, Colorado State University, Fort Collins, CO, USA; 2 GlaxoSmithKline, Neurosciences Therapy Area Unit, Research Triangle Park, NC, USA; 3 Departments of Neurology, Anatomy and Neurobiology, and PMR; University of California, Irvine, CA, USA

**Keywords:** Transcranial magnetic stimulation, Stroke, Cerebral vascular accident, Motor cortex, Clinical trial

## Abstract

**Introduction:**

Transcranial Magnetic Stimulation (TMS) is a neurophysiological tool capable of assessing the motor nervous system and its change over time. In multi-site clinical trials, this technique has some advantages over other neuroimaging methods owing to its relatively low cost, low personnel and equipment infrastructure requirements, and greater ease in consistently applying technology to collect and analyze data. Limited published details exist regarding methods to deliver TMS and analyze data in a standardized and consistent manner as part of an international, multicenter, clinical trial.

**Purpose:**

The objective of this paper is to describe standardized methods of applying TMS motor cortex assessments in an international clinical trial of a pharmacological intervention for stroke patients, which was conducted at 15 centers in three countries.

**Materials and methods:**

A standardization process was developed to ensure TMS protocol adherence and data quality, and each clinical site was required to successfully complete standardization procedures prior to collecting patient data. Key elements of standardization included internet-based training, pilot subject data collection, common TMS equipment across sites, and corrective feedback provided by a standardization administrator. Subsequently, TMS assessments of motor hot spot location, motor threshold, and recruitment curve were conducted in stroke patients on post-stroke Days 5, 30, and 112. Ongoing standardization was maintained by regular review of patient data and communication between the clinical site and standardization administrator.

**Conclusion:**

Although TMS methodological approaches vary, a protocol with standardized procedures was successfully developed and implemented. Using this protocol, centers were formally certified to perform TMS-based neurophysiological measures in this clinical trial of stroke patients. The methodology described is potentially valuable to investigators who might construct future multi-site clinical trials using TMS.

## Introduction

Stroke remains a leading cause of disability for which few therapies have been approved. Approximately 90% of patients survive the initial stroke event, with most survivors experiencing long-term impairments and disabilities. Motor-related deficits commonly result from stroke. In particular, Upper Extremity (UE) hemiparesis has profound effects on daily living activities and functional independence, affecting some 70–80% of survivors of stroke [1]. Unfortunately, only 11% of patients completely recover UE function, and two-thirds experience little to no recovery [2]. These movement-related impairments contribute to disability as well as the very high costs associated with stroke.

To reduce motor-related disability associated with stroke, a number of restorative interventions are under study. Such treatments promote recovery-related neuroplastic mechanisms within the brain [3]. For example, drugs that promote post-stroke axon outgrowth may improve connectivity and activation of surviving neurons to support improved voluntary movement [4]. While behavioral outcome measures are of paramount importance for such interventions, understanding the therapeutic mechanism of action can also be helpful, for example, to inform issues of patient selection and stratification [5], or to serve as a biomarker [6]. A number of different approaches have been suggested for studying the neurological mechanisms of action of restorative therapies. However, challenges exist to implementing many of these techniques in the context of a multi-site clinical trial enrolling human patients with recent stroke. For example, functional imaging has been effectively used to track treatment-related motor system neuroplasticity in single-site intervention research studies, but may be difficult to implement across multiple research sites in a clinical trial for reasons such as cost, need for extensive personnel and equipment infrastructure, differences in approach to data acquisition, and variation in choice of scanning hardware. Transcranial Magnetic Stimulation (TMS), on the other hand, is a neurophysiological measurement tool capable of assessing the motor system and its change over time, and may have advantages in this setting such as lower cost, less personnel and equipment infrastructure requirements, and greater ease in applying consistent technology to collect and analyze data across multiple sites. A key example of this is the report by Sawaki et al. [7] who collected TMS measures across three sites involved in a stroke rehabilitation trial; however this group’s report primarily focused on study outcomes and included limited details regarding their protocols for standardizing collection, management, and analysis of the TMS data. Having a detailed protocol for standardizing key outcome measures is critically important to the conduct of clinical trials, increasing the accuracy of endpoint assessments, reducing variance over time and sites and thus increasing study power, and facilitating data sharing [8–11].

TMS provides a non-invasive means to study the motor cortex. This neurophysiological technique has been used to assess the excitability, and stimulus-response characteristics of the motor cortex of stroke survivors. Several single-site investigations have demonstrated that the excitability characteristics and size of motor cortex representations of the paretic UE change during recovery from stroke or following treatment interventions that target the motor system. For example, motor threshold-a commonly-used TMS measure of global motor cortex excitability-is often abnormally elevated in survivors of stroke, but decreases towards more typical levels as motor recovery occurs [12]. Additional studies have also implicated changes in stimulus-response properties as evidence for changes in neuronal connectivity and recruitment following stroke. Stimulus-response curves or “recruitment curves” assess neurons other than those in the core neuronal population that is activated at motor threshold [13–15]. The threshold for these neurons is higher either because they are less excitable or because they are further from the site of TMS [14]. In survivors of stroke, the initial post-stroke recruitment curve slope is small, i.e., the amount of rise in Motor Evoked Potential (MEP) response amplitude as TMS intensity is increased is relatively small. As recovery progresses, however, recruitment curves tend to become more steep (slope increases), suggesting a neurophysiological strengthening of intracortical and corticospinal connections [16,17].

Groppa et al. [18] recently published general guidelines for using TMS to assess cortical motor threshold and basic MEP characteristics, which provide step-by-step methods for collecting TMS data. In the present study, we sought to expand upon Groppa et al. [18] work by describing a detailed protocol for standardizing a method to collect key TMS outcomes across several international research sites. Specifically, we employed TMS to assess initial and changing neurophysiological motor cortex function in survivors of stroke as part of an international, multi-site, randomized controlled phase IIA trial to evaluate GSK249320, a monoclonal antibody that blocks the axon outgrowth inhibition molecule myelin-associated glycoprotein. A description of the larger clinical trial has been published elsewhere [4]. The purpose of the present manuscript is to detail the protocol critical for successfully implementing TMS in order to assess neurophysiological status and its change in an international, multi-site clinical stroke trial. Novel methodological steps included internet-based training, certification for patient data collection based upon successful pilot subject data collection, common TMS equipment across sites, and corrective feedback provided by a standardization administrator. An ultimate aim of this paper is to detail these methods and experiences gained to inform investigators who might construct future multi-site clinical trials using TMS.

## Materials and Methods

### Overview

The GSK249320 Trial was designed as a multisite, prospective, randomized international clinical trial. Patients were screened, consented, randomized to intravenous GSK249320 or placebo, and received the first infusion within 72 hours of stroke onset. In addition to the primary outcome measure of safety, TMS assessment of neurophysiological status and change was included as a secondary exploratory measure; TMS-based hypotheses examined effects of the drug GSK249320 as well as clinical correlates, and the specific results will reported elsewhere. TMS investigators at each clinical site were masked to treatment condition, as were those analyzing the TMS data. This multi-center study was conducted at 15 centers in three countries (Table 1). Of the 15 centers, 10 were able to enroll at least 1 subject before the trial was completed.

### Subject inclusion and exclusion criteria

Inclusion criteria included stroke onset 24 to 72 hours prior, radiologically confirmed supratentorial ischemic or hemorrhagic stroke, age 18 to 90 years, and sufficient attention and language to participate in study evaluations. Subjects were excluded if any of the following TMS contraindications were present: metal present, such as hardware or plate on the scalp in the area to which TMS was applied, implanted cardiac pacemaker, implanted prosthetic heart valve, medication pump or line, metallic implant or clip in the head/neck, electrical, mechanical or magnetic implants, neuro-stimulation device, or orthodontic work involving ferromagnetic materials; occupation or activity that could cause accidental lodging of ferromagnetic materials or embedded metal fragments in the head; known history of seizures or epilepsy, concomitant use of drugs that substantially lower seizure threshold (e.g., tricyclic antidepressants and neuroleptics); or brain tumor, recent brain injury (within 5 years) associated with definite loss of consciousness, or any history of brain surgery. A total of 42 subjects were enrolled in the trial.

### TMS protocol development

The TMS protocol and its description adhere to guidelines for the conductance and reporting of TMS methods put forth in a recent international consensus study published by Chipchase et al. [19] TMS protocol development was led by two experienced TMS researchers (MM & SC) and took place over a 4-month period beginning in late 2008. These TMS researchers convened several teleconferences with GSK staff to develop the protocol in a manner that would feasibly integrate with all other study components, to form standardization procedures, and to inform TMS-related inclusion and exclusion criteria. TMS outcome measures of motor threshold and recruitment curves were selected based upon their robust ability to provide neurophysiological evidence, and upon the relative ease with which these metrics can be obtained in a standardized way. Motor threshold is commonly employed in TMS investigations of stroke and is a rapidly obtained measure of global motor cortex excitability. Recruitment curves are also commonly applied by TMS investigators in this setting, and the assessment of these generally progresses fairly easily and quickly once motor threshold is obtained. An additional advantage of these metrics is the relative ease and consistency of raw data processing and analysis.

### TMS protocol

**Figure 1:**
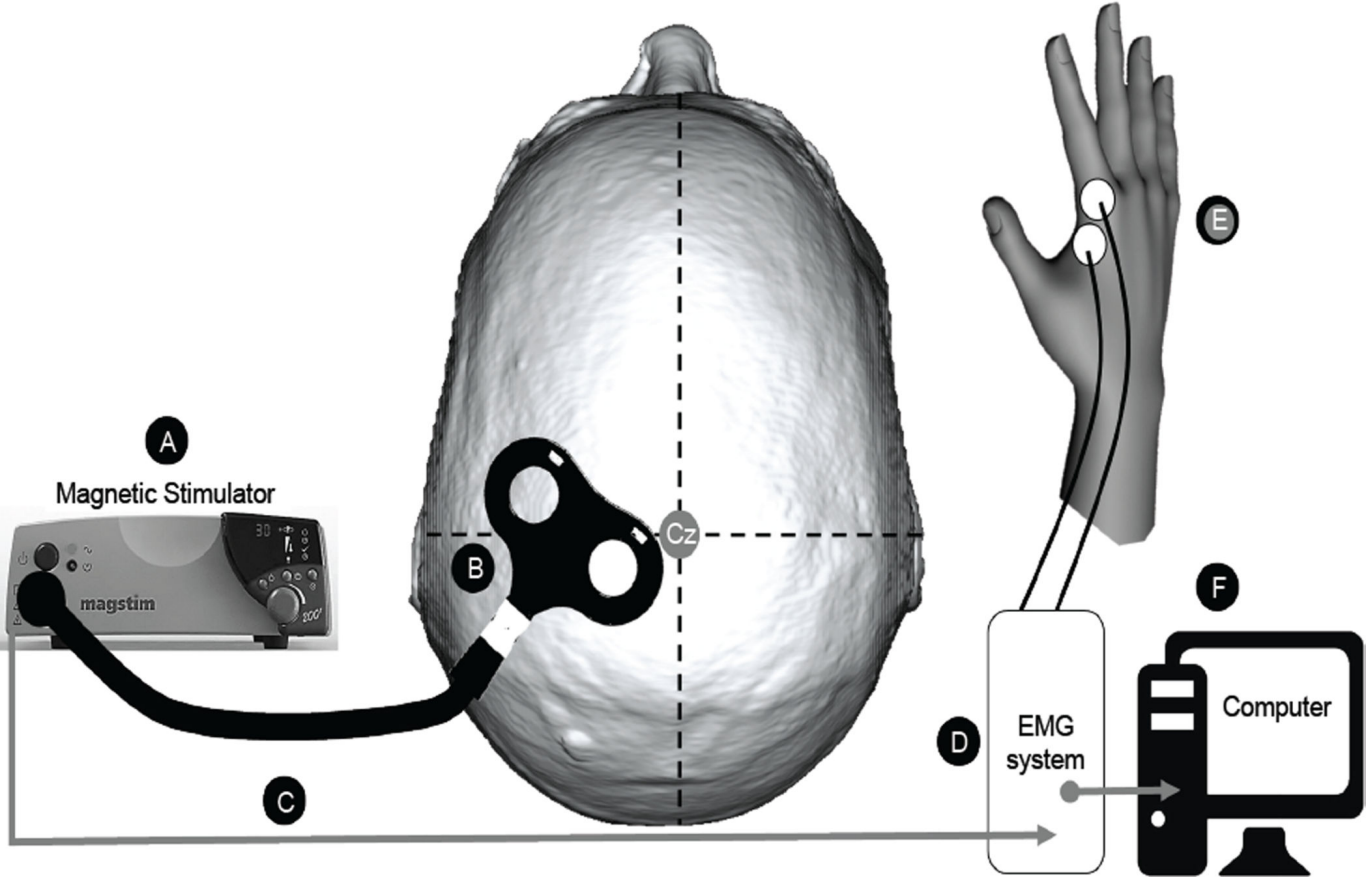
Transcranial magnetic stimulation (TMS) set-up for collecting motor evoked potentials (MEP) **(A)** Magnetic pulses were delivered using a Magstim 200 or 200^2^ magnetic stimulator (Magstim Ltd., Whitland, UK). **(B)** A figure-of-eight shaped coil was oriented at a 45° angle to the mid-sagittal line and approximately 6 cm lateral to the vertex (Cz), such that the coil center rested on the scalp location overlaying the hand representation of the motor cortex in the stroke-lesioned hemisphere. **(C)** Activation of the magnetic stimulator triggered the electromyography (EMG) system **(D)** to record EMG activity from bipolar surface electrodes **(E)** placed over the first dorsal interosseous (FDI) muscle of the hand contralateral to stimulation (i.e., in the hemiparetic hand). Filtered and amplified EMG signals from the EMG were transmitted to a desktop computer **(F)** for further processing and recording.

TMS data were collected on post-stroke Days 5, 30, and 112. Day 5 TMS measures were not performed in subjects that presented with hemorrhagic stroke given the theoretical risk of increased seizure activity early after hemorrhagic stroke. TMS was conducted in a quiet room absent of other equipment that could emit electrical noise resulting in signal contamination. All sites used a Magstim 200 or 200^2^ (Magstim Ltd, Whitland, UK) single-pulse magnetic stimulator to deliver TMS through a 70 mm figure-of-eight shaped magnetic coil. This equipment was loaned to sites by GSK if not already present. Figure 1 displays the TMS set-up for collecting MEPs. The coil handle was oriented 45 degrees from the mid-sagittal line to produce an induced current flow in the anteromedial direction, which is approximately perpendicular to the central sulcus [20] and has been demonstrated as optimal for generating MEPs in intrinsic hand muscles [20,21]. Subjects were seated with the paretic UE supported and bipolar surface electrodes were applied over the paretic first dorsal interosseous (FDI) using a belly-tendon montage and connected to an Electromyograph (EMG). The inter-electrode distance was fixed at 20 mm. A ground electrode was placed over a bony surface of the same arm, typically over the proximal ulna or wrist. To ensure muscle relaxation during the testing session, the TMS technician routinely monitored audio and visual feedback from the EMG. A nylon swim cap was placed on the subject’s head so that stimulation reference marks could be clearly made. To identify the optimal position for stimulation, the stimulator was set at 80% of maximum stimulator output with the subject relaxed. The hand area is often 3–6 cm lateral to the vertex [22]. Multiple sites were sampled in a circle with a radius of 6 cm from this site to define the optimal position, which was defined as the stimulation point that elicited the largest amplitude MEPs. In cases where MEPs were very large (e.g. exceeded the upper gain setting), stimulus intensity was reduced (for example to 70% or lower as needed) so that differences in MEP size were discernible from one stimulating position to the next. Once identified, the location of the optimal position was recorded in relation to its lateral distance (in cm) from the vertex (Cz) and anterior (+cm) or posterior (−cm) distance from the intra-aural line, such that this stimulation location could be replicated across testing sessions. The optimal position was marked on a swim cap worn on the subject’s head.

### Resting motor threshold procedure

The resting motor threshold was obtained with the TMS coil located at the optimal position [18]. We defined motor threshold as the stimulator output that evoked a MEP of at least 50 µV, in at least 5 of 10 trials [23]. Motor threshold was approached from ‘below’, i.e., starting at a low intensity and increasing in 2–5% increments up to threshold. To compensate for possible initial heightened arousal levels and/or startle responses that might affect MEP threshold, several trial stimulating runs were performed prior to the final assessment of motor threshold at the optimal position.

### Recruitment curve procedure

**Figure 2:**
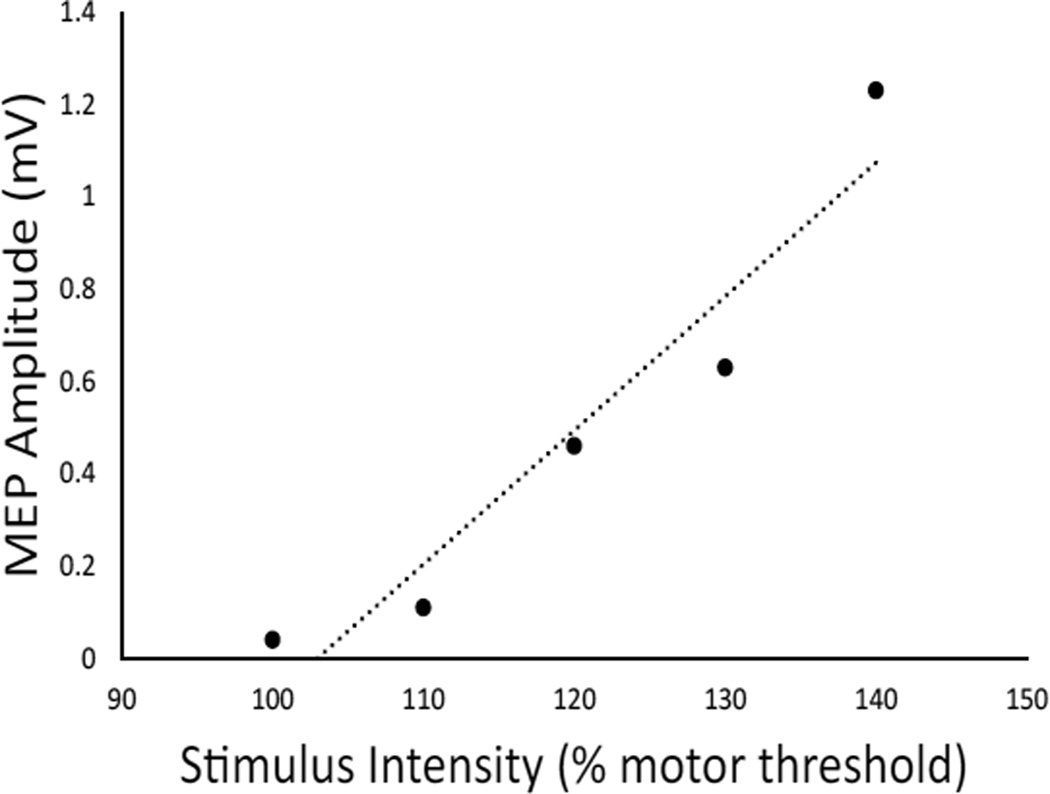
Example recruitment curve from a standardization subject.

The recruitment curve depicts the change in MEP size as a function of stimulus intensity, with input being step (or, TMS intensity, measured as % maximum device output) and output being MEP peak-to-peak amplitude voltage (in mV). Stimuli were applied over the optimal position in steps that varied by 10% of the motor threshold, beginning at the motor threshold value and increasing to 140% of motor threshold, for a total of 5 stimulation steps (i.e., 100%, 110%, 120%, 130% and 140%). Ten stimuli were delivered at each of these steps, with pulses delivered no faster than 0.14 Hz. If a subject was unable to tolerate stimulation to 140% of motor threshold, the maximum tolerable intensity was the effective cut-off point. If a subject had a motor threshold >70% of device output, then some of the steps were not possible because the maximum device output is 100%. For example, if a subject had a motor threshold of 80% stimulator output, only the 100%, 110%, and 120% of motor threshold were the possible stimulus intensities, as the stimulator could not be set to 104% output to achieve the 130% of motor threshold. A linear fit of recruitment curve data was used to generate the primary curve metric of slope. Figure 2 displays an example recruitment curve obtained in a standardization subject.

**Figure 3:**
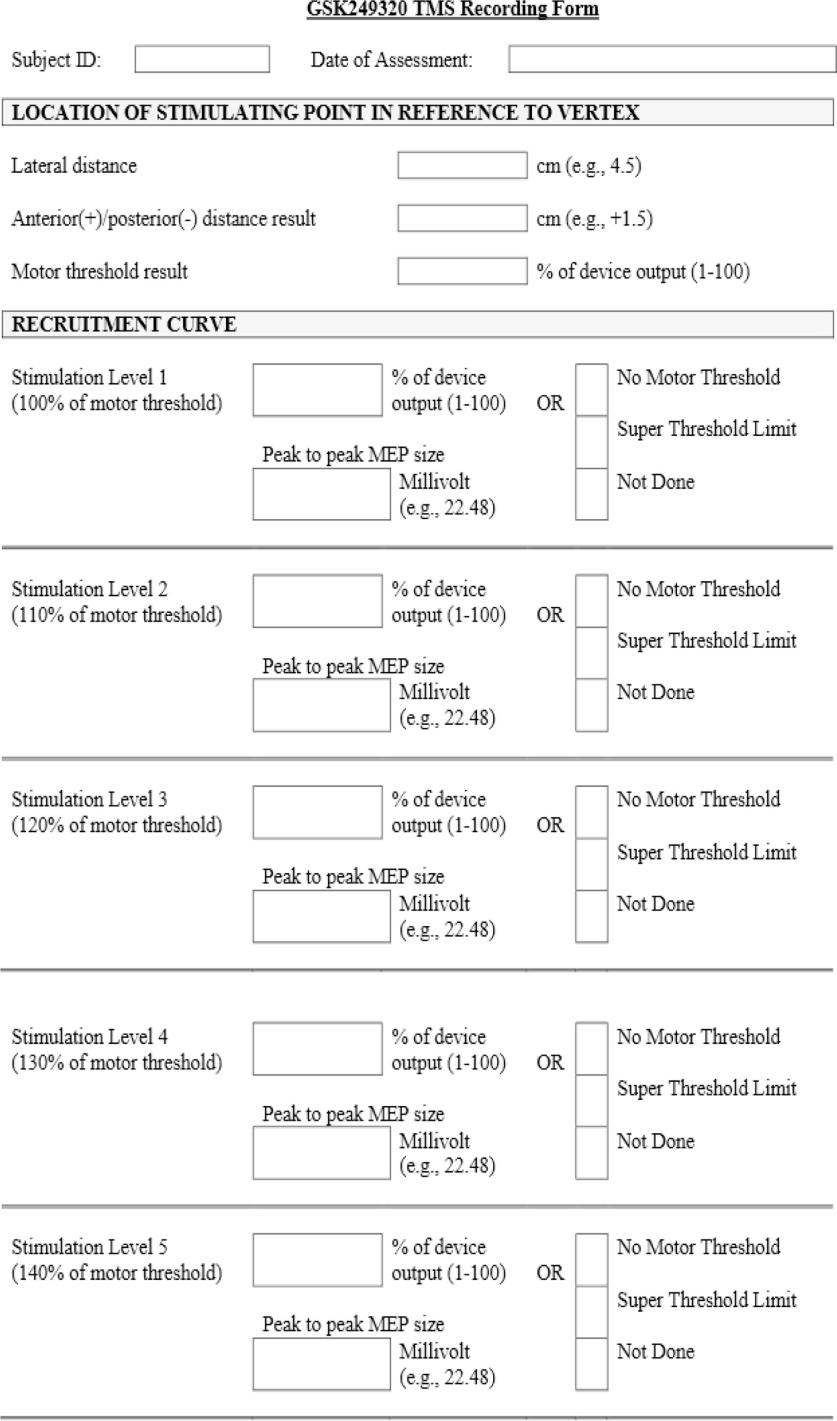
TMS recording form.

For each recruitment curve stimulation intensity, a mean MEP response of the 10 trials was obtained using online averaging through the EMG software. Measurement of the mean MEP amplitudes [18] was conducted at the respective site and electronically submitted to the data management center along with motor threshold and optimal position location. The most common variable to affect MEP visualization is gain (magnification). As such, investigators were instructed to maintain the same display gain in the EMG software during MEP measurement across trials and testing sessions for each subject. All TMS testing and outcome data were recorded on the TMS Data Collection form (Figure 3).

## Standardization

**Figure 4:**
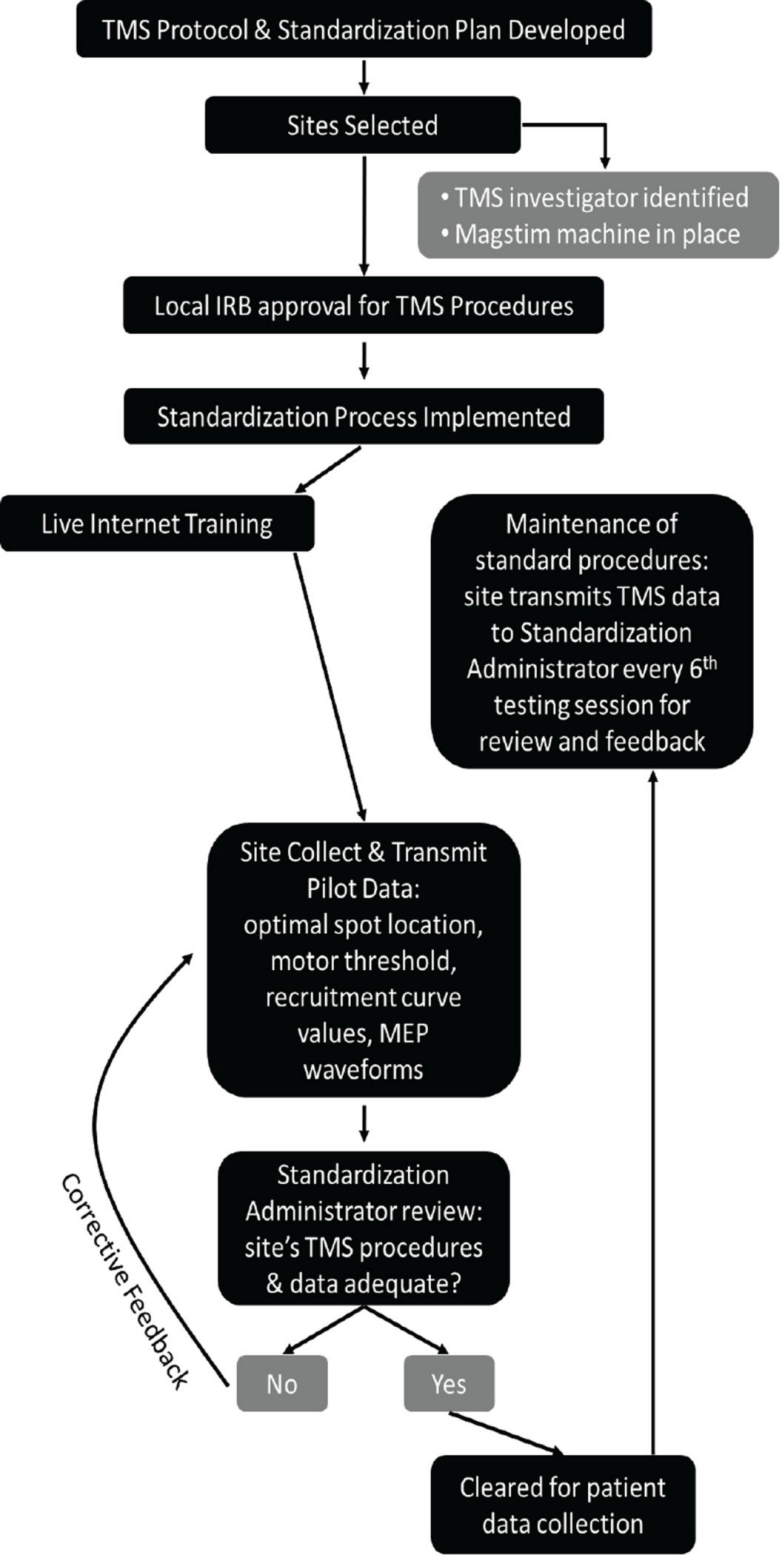
Flow chart of TMS protocol development and standardization procedures.

The standardization procedure included initial and maintenance standardization processes overseen by a standardization administrator experienced in TMS procedures (Figure 4). The intent of such standardization was to ensure that both the collection and the analysis of TMS data were performed in a manner consistent with the TMS protocol, across collection sites, and over time. Clinical sites were required to have an experienced TMS investigator. Prior to performing any TMS-related study procedures, each site participated with a live internet-based training session, led by the standardization administrator, which provided verbal, written, and pictorial details on the TMS testing schedule, TMS-related risk management, TMS protocol, data analysis, and standardization procedures.

At each site, prior to enrolling the first patient into the clinical trial, pilot data were collected on 1 or 2 neurologically intact individuals in accordance with the TMS protocol. Clinical sites were provided with example data to guide their own data collection. Assessments carried out as part of initial standardization were finding the optimal stimulating position, motor threshold, and recruitment curve (Figure 4). Optimal position location, motor threshold intensity, and averaged MEP amplitudes at each recruitment curve intensity were recorded on the TMS standardization form and transmitted to the standardization administrator along with MEP waveforms. The standardization administrator specifically examined the data to ensure that each of the following 7 points was addressed:

1. Pilot subject ID and date of assessment were recorded.
2. Optimal stimulating point coordinates were correctly recorded (relative to vertex).
3. Motor threshold was recorded as % of device output.
4. Stimulation intensity #1 during the recruitment curve procedure was equal to the documented motor threshold and each subsequent recruitment curve intensity was correctly set relative to the motor threshold and at the correct % of motor threshold.
5. Peak-to-peak MEP size (amplitude) was recorded in mV for each recruitment curve intensity, or else a satisfactory explanation was provided explaining why MEPs could not be collected.
6. The standardization administrator discussed any deficiencies in the recording of data or stated problems/questions with the clinical site via phone or email.
7. Waveforms during the recruitment curve procedure were examined for common issues seen in TMS data collection (Table 2) and corrective actions were discussed between the standardization administrator and clinical site.

**Figure 5:**
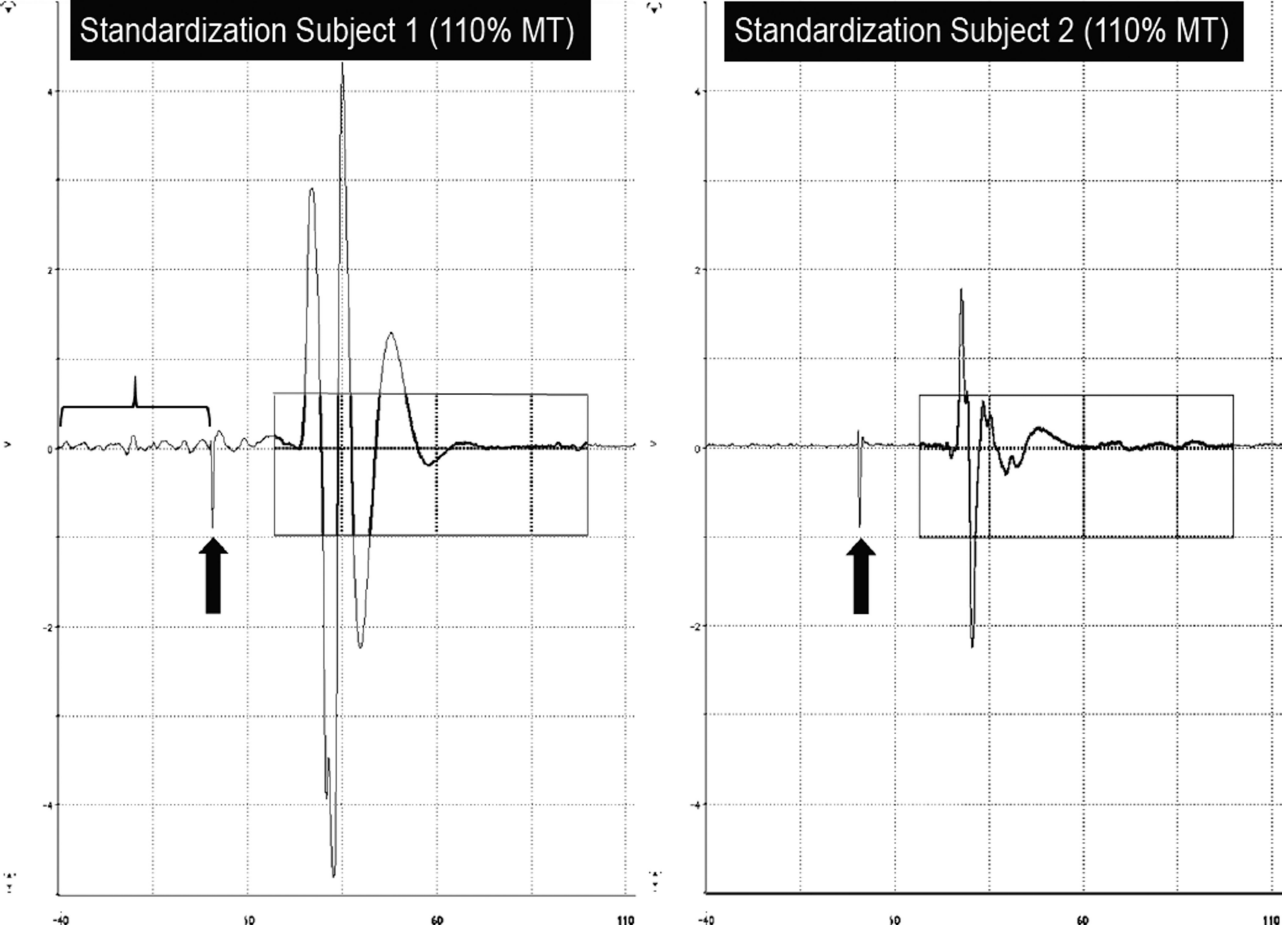
Example standardization waveforms from a clinical site For Standardization Subject 1 at this clinical site, the target muscle was not sufficiently relaxed (bracketed region of EMG record) prior to TMS onset (black arrow). Such pre-stimulus muscle activity appears to have facilitated the MEP, as evidenced by its rather large amplitude. Following feedback to monitor baseline EMG activity for target muscle relaxation, a second standardization subject (right panel) was run and demonstrated a much quieter EMG pre-stimulus baseline. For both subjects, these example MEPs were collected with the TMS stimulator set to 110% of the resting motor threshold (MT). NOTE: the boxed region represents the time window in which the MEP amplitude was measured.

When deficiencies or problems arose during the initial TMS standardization, in most cases, the clinical site was asked to collect and submit data from an additional pilot subject. Figure 5 for example MEP waveforms obtained during the standardization process, which assisted the clinical site in taking corrective action to ensure pre-stimulus muscle relaxation. When the standardization administrator was satisfied that the data collected by the site met all 7 criteria, the site was formally certified for collecting TMS data in this clinical trial. All 15 clinical sites successfully performed all standardization procedures and were certified. All TMS data were collected between July 2009 and January 2011 and were from certified sites.

To maintain adherence to standardized procedures throughout the study, each site was asked to transmit the standardization documents on the first patient assessment collected in the study and then on every 6^th^ assessment performed thereafter. The standardization administrator reviewed these data and communicated with the site about any necessary corrections in the collecting or analysis of TMS data.

## Adverse Events

The TMS protocols used in this trial followed published safety guidelines [24]. Although the occurrence of potential side-effects associated with single-pulse TMS are rare, we took necessary steps to further reduce their likelihood of happening. Two potential serious side effects of TMS are seizure and interference with implanted electrical devices [24], and as such, the clinical trial excluded individuals who had a history of seizures or epilepsy, or had an implanted electrical device. Investigators were responsible for detecting, documenting and reporting events that met the definition of an Adverse Event (AE) or Serious Adverse Event (SAE). AEs and SAEs were collected from start of investigational product on Day 1 through the end of a subject’s study participation and were recorded in the GSK electronic case report form. A GSK data review committee regularly reviewed all AEs and SAEs.

## Discussion

While clinical trials suggest that restorative therapies can promote motor recovery after stroke when paired with rehabilitation [25–27], limited information is available regarding the neural mechanisms of action for such interventions in humans. A handful of multi-site clinical trials have incorporated such measures, and among these TMS assessments have not been a common choice, owing in part to challenges of delivering then implementing a standardized protocol. The aim of the current report is to define key factors and methodological details required to consistently administer TMS as a neurophysiological measurement tool across multiple clinical trial sites. The primary methodological innovation around our application of TMS was the procedure for standardizing the TMS protocol and for certifying sites as trial-ready. Key components of this standardization included: (1) internet-based training on TMS procedures, (2) initial collection of standardization data in neurologically intact pilot subjects, (3) thorough review of initial and maintenance standardization data by an standardization administrator experienced in TMS, (4) frequent communication and immediate problem solving between the standardization administrator and clinical sites, and (5) codification of training through a formal certification process. An additional important feature of applying consistent methods across sites was the use of the same make and model of magnetic stimulator. In this study, sites were equipped with either the Magstim 200 or the second generation Magstim 200^2^, which both produce a monophasic pulse. In contrast to biphasic stimulators, monophasic stimulators produce a pulse output with greater spatial accuracy and lesser chance for overheating [28].

Although motor threshold and recruitment curves are commonly acquired measurements in TMS research, investigators show substantial variability in the specific details employed in the acquisition and extraction of these TMS metrics. For example, numerous approaches have been published for locating the optimal stimulating position. We stimulated over a relatively focused area surrounding the typical “hot spot” location for activating the motor cortex representation for contralateral intrinsic hand muscle activation. Other methods for locating the optimal position include stimulating a larger area of cortex or use of frameless stereotaxic system. We elected to use a more conservative stimulation area to maximize time efficiency and to reduce the overall number of stimulations to which the subject was exposed. The standardized TMS protocol did not require clinical sites to use a frameless stereotaxic system. While such a method does enhance the physiologic effect of TMS [29], and would arguably further improve the test-retest reliability of TMS [15], use of the equipment in clinical settings may be prohibitive given time, space, and portability limitations.

In generating recruitment curves, Malcolm et al. [15] advocate for use of a sigmoid mathematical function to characterize recruitment curve data, which at least theoretically approximates an S-shaped curve. To apply a sigmoid function in characterizing TMS recruitment curve data, a minimum of four (4) abscissa points, i.e., stimulation intensity values, are required. While a sigmoid fit has advantages, the high threshold seen in stroke patients translates to many individuals potentially approaching a suprathreshold limit at higher recruitment curve intensities (e.g., 130%, 140% of motor threshold), thereby reducing the number of available data points. For this reason, characterizing recruitment curves favors a linear fit for this population. Certainly, using a greater number of stimulation intensities would add greater detail to the recruitment curve, but would also significantly increase the length of the assessment period and exposure to magnetic stimuli.

Variations exist in how investigators visualize and measure MEPs during raw data processing and analysis. In this study, investigators were instructed to use a consistent display gain during offline measurement of MEPs across trials and testing sessions. While some TMS studies measure the area-under-the-curve of rectified MEPs, the majority of investigations calculate MEP size based upon the peak-to-peak amplitude. We used the latter approach to measuring MEPs, as the MEP offset is sometimes difficult to distinguish from baseline EMG activity in area-under-the-curve measurements.

This study’s standardization and certification procedures ensured good data quality, and that a common TMS protocol was used across sites and patients during subject preparation, testing, and data analysis. Other contributions that helped to ensure consistent and valid TMS procedures included protocol development and delivery by experienced TMS investigators; communication between the scientists developing the TMS protocol and other study scientists and administrators to ensure appropriate integration with the larger clinical trial; routine evaluation of TMS data entered during the trial; electronic case report form and data management practices to screen for data entry errors; communication between the standardization administrator and TMS investigators at each site when questions or problems arose about data validity and quality during standardization procedures and trial data collection; and blinding of TMS investigators and data analyzers to intervention assignment.

## Conclusion

Despite the wide variety of methodological approaches employed across sites engaged in TMS research [30], the GSK249320 trial was able to successfully incorporate a standardized protocol to train, certify, and then employ TMS as a neurophysiological measure in a phase IIA trial of stroke patients. The procedures to maintain standard methods and data checking assured high quality data and data integrity. The TMS protocol adhered to strict risk-management and safety methods, which likely contributed to the absence of any TMS-related adverse events. No such standardization method has been described to date, therefore, the design and implementation of our standard approach opens the door for including TMS assessment of neurophysiological status and change in future multi-center clinical trials.

## Figures and Tables

**Table 1:** Clinical Sites^a^.

Country	Hospital/Institution, City, State/Province
Canada	Sunnybrook Health Sciences Centre, Toronto, ON
	Jewish General Hospital, Montreal, QU
Germany	Universitaet Essen, Essen
	Dr. Horst-Schmidt-Kliniken GmbH, Wiesbaden^b^
	AKH Celle Neurologische Klinik, Celle
	Universitaetsklinik Ulm, Ulm
	Universitaetsmedizin der Johannes Gutenberg-Universitaet, Mainz^b^
	Universitaetsklinikum Leipzig, Leipzig
	Universitaetsklinikum Koeln Klinik und Piliklinik fuer Neurologie, Koeln^b^
	Medizinische Hochschule Hannover, Niedersachsen
United States	University of California Irvine Medical Center, Orange, CA
	Oregon Health and Science University, Portland, OR
	Wayne State University & Detroit Medical Center, Detroit, MI^b^
	Poudre Valley Hospital & Colorado State University, Fort Collins, CO
	Ronald Reagan UCLA Medical Center, Los Angeles, CAb

a The clinical trial, including investigational product management, was monitored by GlaxoSmithKline (GSK). Subjects were registered and randomized using an Interactive Voice Response System (IVRS). Data management and statistical analysis were handled by GSK. Standardization of TMS procedures was managed at Colorado State University.

b Screened but did not enroll patients.

**Table 2:** Potential issues and corrective actions for recruitment curve data collection.

Issue	Corrective action
Incorrect marking of MEP peak and trough.	The clinical site was provided with further examples of correct marking of MEPs.
Presence of excessive ambient (50 Hz or 60 Hz) noise in the signal.	The clinical site was requested to check electrode placement, ground placement, and for the presence of non-study electrical equipment that could be powered down to eliminate electrical noise.
Presence of excessive stimulus artifact.	The clinical site was requested to try different positions of the ground electrode.
Presence of excessive background EMG activity.	The clinical site was requested to provide proper support and positioning of the target UE.
